# Protective Role of Gallic Acid Against Corticosterone-Induced Hepatic Toxicity: Modulation of Oxidative Stress and Inflammatory Pathways in Wistar Rats

**DOI:** 10.3390/toxics13100897

**Published:** 2025-10-20

**Authors:** Priyanka Tiwari, Prabhat Kumar, Saripella Srikrishna, Nikhat Jamal Siddiqi, Bechan Sharma

**Affiliations:** 1 Department of Biochemistry, Faculty of Science, University of Allahabad, Prayagraj 211002, India; priyankabhu757@gmail.com; 2 Department of Biochemistry, Institute of Science (BHU), Varanasi 221005, India; prabhatbiochem@bhu.ac.in (P.K.); skrishna@bhu.ac.in (S.S.); 3 Department of Medical Surgical Nursing, College of Nursing, King Saud University, Riyadh 11421, Saudi Arabia; niksiddiqi@gmail.com

**Keywords:** corticosterone, gallic acid, liver toxicity, oxidative stress, hepato-protectant

## Abstract

Corticosterone (CORT), a key stress hormone, is vital for energy balance, but prolonged exposure causes hyperglycemia, obesity, and hepatotoxicity. Gallic acid (GA), a natural polyphenol with antioxidant and anti-inflammatory properties, was evaluated for its hepatoprotective effects in Wistar rats. This study aimed to assess how GA protects against CORT-induced liver toxicity in Wistar rats and to explore its molecular interactions through in silico docking studies. Animals received CORT (15 and 30 mg kg^−1^ body weight) orally for 21 days, with GA pretreatment in selected groups. Hepatic status was assessed via biochemical assays, molecular markers, histopathology, and in silico docking. CORT significantly increased body weight (15%), blood glucose (1.5-fold), malondialdehyde (MDA; 28%), and protein carbonyls (34%,) with a statistical significance, *p* < 0.05 and <0.01, while glutathione (41.4% to 52.1%) and antioxidant enzymes were significantly reduced (statistical *p*-value significance at levels of <0.05, <0.01, and <0.001). GA pretreatment restored glucose MDA, and GSH toward control (*p* < 0.01), and protected histological injury. Docking studies showed strong GA binding to Keap1 (−6.9 kcal/mol), IKKβ (−6.0 kcal/mol), and COX-1 (−6.2 kcal/mol), supporting its antioxidant and anti-inflammatory action. GA confers significant protection against CORT-induced hepatotoxicity, validated by both in vivo and in silico analyses.

## 1. Introduction

Glucocorticoids (GCs) regulate homeostasis in response to the stressful conditions of an organism. After the discovery of synthetic steroids, these molecules (steroids) came into the limelight to cure inflammatory conditions as the first therapeutic choice by clinicians [1,2]. Steroidal drugs are widely prescribed for managing arthritis, asthma, and autoimmune diseases in India. Long-term therapy with steroidal drugs, especially at high doses, elicits several side effects, including oxidative stress, obesity, weight gain, insulin insensitivity, and a higher risk of developing diabetes mellitus [3,4,5,6,7,8,9]. However, some side effects might be reversed using supplements or prophylactic agents, or with the discontinuation of the therapy [10,11,12,13].

Exposure to GCs disrupts glucose metabolism and homeostasis, which has been extensively reported in both human and rodent models [14,15]. This disruption may have direct or indirect influences at various locations [16]. The direct effects of GCs encompass a wide range of metabolic outcomes, such as an increase in liver gluconeogenesis, insulin suppression, and hindrance in glucose absorption by peripheral tissues, leading to the release of stored energy and its redirection to muscles [15,17,18,19,20]. Alterations in physiological levels of GCs are also linked to behavioral changes, including the suppression of reproductive functions, heightened anxiety, and modifications in aging and feeding patterns [21,22]. The activity of GCs on the neuronal tissues contributed to a detrimental impact on glucose metabolism and insulin levels [23,24].

The exposure of GCs to mammals causes lipid peroxidation, which results in a significant decrease in hepatic nonenzymatic and enzymatic antioxidants [25,26,27,28], heart and spleen [29], erythrocytes [30], skeletal muscle, renal tissues [27], and lymphoid organs [31] of the rat. Harno et al. have demonstrated the effects of GCs in rat liver, causing hepatic steatosis and adversely influencing insulin homeostasis via Agouti-related protein (AgRP)-expressing neurons [10]. Recently, an observation recorded by Wu et al. has indicated that chronic exposure to GC may disrupt the hepatic and intestinal bile acid metabolism, resulting in fatty liver development in chickens [32].

At the molecular level, the effects of GCs have been shown to involve the inhibition of the synthesis of cytokinins such as interleukins, interferon (IFN), granulocyte macrophage-colony stimulating factor (GM-CSF), and tumor necrosis factor (TNF)-α via modulation of transcriptional, translational, and secretory processes [33,34]. However, not all the cytokinins are inhibited by the corticosteroids. Interleukin-4 (IL-4), a type I cytokine with a four-α-helical bundle, encompasses pleiotropic actions on multiple lineages [35]. Earlier reports have demonstrated perturbations in the levels of IL-4 due to the introduction of GCs [36]. IL-4 is commonly known to control allergic reactions, inflammation, immunoglobulin production, fibrosis, and antitumor activity [37,38].

The glucocorticoids were also found to induce apoptosis of lymphocyte tissues [39]. Siegel and their colleague have indicated the development of resistance in the thymocytes due to GC-induced apoptosis in Bcl-2 transgenic mice [40]. However, GC’s actions on procaspase expressions are not well studied. Procaspase-3 (PC-3) is a precursor of caspase-3, with a pro-domain, a large subunit, and a small subunit. Proteolysis at Asp9, Asp28, and Asp175 of the PC-3 regions results in caspase-3 activation. The overexpression of PC-3 has been reported in different cancer types. Recent studies have suggested that an elevation in sub-apoptotic caspase-3 activation may lead to oncogenic transformation, which might be related to the overexpression of PC-3. The mechanism behind this is still not well understood. However, understanding PC-3 activators might be useful in anticancer therapy [41].

Natural compounds have been found to act as protective agents against oxidative stress and liver injury generated by heavy metals and drugs in mouse models [42]. It has been reported that the implication of some phytochemicals having antioxidant and anti-inflammatory properties might be useful in suppressing GC-mediated adverse effects [27].

Gallic acid (GA, 3,4,5-trihydroxybenzoic acid), a polyphenol compound predominantly present in various plant products, acts as an antioxidant [43], anti-inflammatory, anticancer, cardioprotective, gastroprotective, and neuroprotective agent [44]. It is also effective against metabolic syndrome [45], and liver inflammation induced by a mutagen, N′-Nitrosodiethylamine (NDEA), in Wistar rats [46]. A recent study showed the pretreatment of GA against t-BHP-induced hepatotoxicity in L02 cells, indicating its hepatoprotective properties [47]. In a recent study, it was also reported that GA is an important component that regulates the KEAP1/NRF2/ARE pathway by triggering its dynamic binding to KEAP1 [48].

A thorough review of the literature indicated that the toxic effect of CORT on the liver was not critically investigated, and no attempt was made to evaluate the protective effect of GA in CORT-mediated toxicity in mammalian systems. The current study was therefore carried out to assess the impact CORT on the cellular organization, biochemical, molecular, immunological, and apoptosis parameters, and the protective effect of GA in CORT-treated rat liver. The results suggested that the long-term implication of CORT significantly altered the antioxidant status, hepatic marker enzymes, and elevated levels of proapoptotic markers like procaspase-3 and IL-4. The prior administration of GA was able to reverse the alteration in oxidative stress parameters and liver function enzymes. The protective property of GA against CORT-mediated toxicity could open a new vista to explore its pharmacological potential.

## 2. Materials and Methods

### 2.1. Chemicals

Analytical grade solvents and chemicals were used in this study. Corticosterone (CORT) and H_2_O_2_ (TCI Chemicals Pvt. Ltd., Toshima, Kita-KU, Tokyo, Japan), gallic acid (Himedia Pvt. Ltd, Mumbai, Maharashtra, India), sodium carbonate, sodium dihydrogen phosphate, sodium hydrogen phosphate, NaOH, NADH, EDTA, and DNPH were purchased from MERK. 1-chloro-2, 4-dinitrobenzene (CDNB), reduced glutathione, Folin–Ciocalteu reagent (FCR), sodium pyruvate, and bovine serum albumin (BSA) (SRL Pvt. Ltd., Mumbai, India), Hematoxylin and Eosin, DAPI were purchased from MERCK. The ATP kit (Sigma-Aldrich, St. Louis, MO, USA), ECL kit (Bio-Rad, Hercules, CA, USA), PVDF membrane (Millipore, Bedford, MA, USA), and antibodies (Invitrogen, Carlsbad, CA, USA).

### 2.2. Habitat of the Animal

The male Wistar rats, ranging from 100 to 150 g, were purchased from the CDRI India. The rats were kept under standard laboratory conditions (three animals/cage; six animals/group; 25 ± 2 °C, with a relative humidity of 55–64%) with a natural light/dark cycle (12 h). There is free access to deionized water and standard food for the animals. All experiments were conducted in accordance with the recommendations and regulations of the University of Allahabad Institutional Animal Ethical Committee (IAEC).

### 2.3. Ethical Clearance

All experiments were conducted under the guidelines established by the Institutional Animal Ethical Committee (IAEC) at the University of Allahabad (IAEC Approval no. IAEC/UoA/012/2022). The 3R principle was followed for all experiments. The experimental animals were handled carefully and anesthetized properly to ensure minimal distress to the animals.

### 2.4. Experimental Protocol

All animals underwent a period of acclimatization to controlled laboratory settings and were observed daily for a week before the commencement of experimental procedures. During this timeframe, the rodents were allocated randomly to groups containing six rats in each, ensuring similar weights within each group (*n* = 6).

The sample size was chosen based on previous studies in similar models and was sufficient to detect significant changes in the measured outcomes. Animals were randomly allocated to groups. All experimental treatments were performed in a blinded manner, and the investigators analyzing biochemical, histological, and molecular outcomes were unaware of the group allocation until after statistical analysis.

The CORT (sourced from TCI, Chennai, India) solution was prepared using physiological saline, 0.9% (*w*/*v*; with a final concentration of 2.5% absolute ethanol), and administered orally via gavage to the rats at dosages of 15 and 30 mg CORT per kilogram of body weight per day. GA was dissolved in deionized water and given to the rats (50 mg kg^−1^ of body weight) accordingly. The control group of rats (C) was treated with physiological saline, 0.9% (*w*/*v*), containing a final concentration of 2.5% absolute ethanol.

The treatments were given according to the experimental design presented below.

Group (1) Control (C)

Group (2) GA treatment [50 mg kg^−1^]

Group (3) CORT treatment [E1,15 mg kg^−1^]

Group (4) CORT treatment [E2, 30 mg kg^−1^]

Group (5) GA + E1

Group (6) GA + E2

At the end of the treatment period, the rats from all groups were euthanized and sacrificed; the blood was collected in sterilized tubes, followed by removal of liver and other tissues, and stored at −20 °C.

### 2.5. Behavioral Analysis and Body Weight Monitoring

Animals were assessed by manual observation throughout the dosing period (daily) for behavioral changes, including locomotion, feeding habits, water intake, aggression, etc. A semi-quantitative scale was used to assess the severity of alterations, where ‘+’ indicated mild changes, ‘++’ moderate changes, and ‘+++’ severe changes. The body weight of each animal was recorded weekly using a digital scale (Citizen Scale (i) Private Limited, Mumbai, India) throughout the experimental period to evaluate the effects of CORT and GA treatment on overall health and weight of the animals.

### 2.6. Collection of Blood Serum

Blood was taken from the individual animal with a cardiac puncture and kept in sterilized tubes at room temperature. The clotted blood was centrifuged using a Sigma bench-top refrigerated centrifuge (Model no. 3-18 K, Goldbach, Germany) at 1500× *g* for 30 min to obtain serum.

### 2.7. Preparation of Tissue Homogenate

The animals were sacrificed, the liver was removed and washed with ice-cold saline (0.9% NaCl), dried using blotting paper, and weighed. The 10% (*w*/*v*) homogenate was prepared with chilled 0.25 M sucrose solution under ice-cold conditions (4 °C), and a Potter-Elvehjem homogenizer (DWK Life Sciences, Mainz, Germany), fitted with a Teflon-coated pestle, was used for this purpose. The homogenate obtained was centrifuged at 9000× *g* for 30 min, and the supernatant was collected in separate aliquots to be used as fresh or stored at −20 °C for further use.

### 2.8. Protein Assessment

The protein concentration in different samples was determined employing the method of Lowry et al., using bovine serum albumin (BSA) as a standard [49].

### 2.9. Assessment of Nonenzymatic Antioxidant Status in Rat Liver

#### 2.9.1. Glutathione Content (GSH) in Rat Liver

The Ellman method [50] was used to quantify the GSH content in hepatic tissue homogenate. The optical density of the supernatant of the yellow color complex was measured at 412 nm. An incubation period of 15 min with intermittent shaking was employed for a reaction mixture comprising 200 mM Tris-HCl buffer, pH 8.2, 0.01 M DTNB, and 200 µg protein, followed by centrifugation at 800× *g* for another 15 min. The findings were reported in µg of GSH/mg protein, with the GSH standard for the calculations.

#### 2.9.2. MDA Analysis in Rat Liver

To detect lipid peroxidation in the cellular fraction of the rat liver, the malondialdehyde (MDA) level was measured using Niehaus and Samuelsson’s method, and the results were expressed as nmol MDA mg^−1^ protein (1.56 × 10^5^ M^−1^ cm^−1^ = molar extinction coefficient) [51].

#### 2.9.3. Estimation of Protein Carbonyl Content in Rat Liver

The PCO contents were determined using DNPH following the method outlined by Kumar et al. [52]. The liver homogenate (200 µg protein) was incubated with 10 mM DNPH prepared in 2 M HCl. A reference set was prepared using the tissue homogenate without DNPH. An equivalent volume of 10% (*w*/*v*) TCA was added to precipitate the protein, and centrifuged at 3400× *g* for ten min; the resulting pellet underwent treatment with ethanol and ethyl acetate (1:1, *v*/*v*) for the elimination of additional DNPH, and then dissolved in guanidine hydrochloride (1.25 mL, 6 M). The PCO content was evaluated by taking the absorbance at 370 nm.

### 2.10. Determination of Oxidative Stress Status in Rat Liver

The standardized, sensitive, and rapid procedures used to optimize oxidative stress parameters in the rat liver.

#### 2.10.1. Estimation of Superoxide Dismutase (SOD) Activity in Rat Liver

The activity of SOD was assayed by using a 20 µL sample (150 µg protein), and the inhibition of pyrogallol auto-oxidation in 50 mM Tris succinate buffer (pH 8.2) was monitored spectrophotometrically at the wavelength of 420 nm. The required amount of enzyme at which 50% inhibition of pyrogallol auto-oxidation was found was considered as one enzyme unit [53].

#### 2.10.2. Estimation of Catalase (CAT) Activity in Rat Liver

The activity of CAT was measured in 20 µL of rat homogenate (150 μg protein) in 50 mM phosphate buffer (pH 7.0). The absorbance decrement at 240 nm wavelength was recorded due to H_2_O_2_ (30 mM) decomposition. The amount of enzyme decomposing 1 mM H_2_O_2_ per min was defined as one enzyme unit [54].

#### 2.10.3. Estimation of Glutathione S-Transferase (GST) Activity in Rat Liver

The GST activity was assayed by using 200 mM phosphate buffer (pH 6.5), 25 µL rat liver homogenate, CDNB (1 mM), and GSH (1 mM) in the reaction mixture. The rise in the absorbance at 340 nm was monitored using a UV-visible double-beam spectrophotometer (Thermo Fisher Scientific, Waltham, MA, USA). The amount of enzyme catalyzing the formation of 1 mM product per minute under specific assay conditions was defined as one unit of enzyme activity [55].

### 2.11. Oxidative Stress Index (OSI)

The OSI was estimated using the following formula [42,56].

(1) $$\mathrm{OSI}=\frac{level\;of\;oxidant(MDA)}{levels\;of\;antioxidant\;enzymes\;SOD+CAT+GST}$$

The units were normalized to protein content before calculation to ensure unit consistency and comparability across groups.

### 2.12. Quantification of ATP in the Liver of Rats

The ATP was quantified using the procedures of an ATP assay kit with luciferin and luciferase (TA100, Toyo B Net, Tokyo, Japan). The liver tissue was extracted in the ATP extraction buffer. Briefly, minced liver tissues (about 0.1 g) were washed with PBS, resuspended in ATP extraction reagent, and then centrifuged at 10,000× *g* for 10 min. The supernatant was used for the ATP assay. The 50 μg of protein was taken for further evaluation.

### 2.13. Determination of the Level of Hepatic Function Enzymes in Rat Blood Serum

#### 2.13.1. Assays for the Activities of Liver Transaminases Alanine Aminotransferase (ALT) and Aspartate Aminotransferase (AST)

The alanine aminotransferase (ALT) and aspartate aminotransferase (AST) quantification in the serum were determined by the previously described methodology [57]. For ALT assessment, the protein specimen was introduced into a buffered solution of DL-alanine and α-ketoglutarate (pH 7.4) and underwent incubation at 37 °C for 30 min. Subsequently, DNPH (1.0 mM) was introduced after the incubation period, followed by adding 0.4 M NaOH, with absorbance measurements taken at 510 nm. In the case of AST evaluation, the protein sample was combined with a buffered solution comprising α-ketoglutarate and L-aspartic acid (pH 7.4) and underwent incubation for an hour at ambient temperature. After incubation, DNPH (1.0 mM) and NaOH (0.4 M) were added. The absorbance was recorded at 510 nm.

#### 2.13.2. Assays for the Estimation of Alkaline Phosphatase (ALP) and Acid Phosphatase (ACP) Activity in the Serum

The activities of alkaline phosphatase (ALP) and acid phosphatase (ACP) were measured using standard protocols [58]. The absorbance change was recorded using a UV-visible double-beam spectrophotometer (Thermo Fisher Scientific, USA) at 405 nm. The reaction mixture without enzyme was taken as a reference, and the activity of ALP was denoted in IUL^−1^.

### 2.14. Estimation of Lactate Dehydrogenase (LDH) Activity in the Serum

LDH activity in serum was assessed through a well-known protocol [59]. The assessment measured the reduction in NADH absorbance at 340 nm over a 3-min duration. The reaction mixture was prepared using Tris-HCl buffer (67 mM, pH 7.4), KCl (33 mM), sodium pyruvate (17 mM), NADH (800 µM), and appropriately diluted protein to reach a volume of up to 3 ml. The absorbance reduction was observed at 340 nm over 3 min, and enzyme activity was calculated. The control for this assay system was the reaction mixture without homogenate. The specific activity of LDH was quantified as µmoles min^−1^ mg^−1^ of protein.

### 2.15. Estimation of Glucose Concentration in Serum and Evaluation of Body Weight Change

The glucose level in serum was measured using the Eco-Pak glucose assay kit protocol, keeping glucose as a standard. The 5 µL sample (30 µg protein) and 99 µL of the Eco-Pak glucose assay reagent were mixed and kept for 30 min at 37 °C. The absorbance was taken at 500 nm wavelength using a multimode plate reader (Synergy H1 Biotech). The daily weight of the mouse was measured using a digital balance, and the weekly average weight was calculated to determine the percent change in body weight.

The liver homogenate was prepared as described earlier. Protein concentration was measured using the Bradford method (Synergy H1 microplate reader, BioTek, Winooski, VT, USA). The protein (30 μg) was diluted with an equal volume of 2X protein loading dye and heated for 10 min at 70 °C in a water bath. Samples were cooled and loaded into a 10% SDS polyacrylamide gel. The SDS-PAGE was performed at 50 V for 3 h in a running buffer. Gel was then transferred to PVDF membrane (Millipore, Bedford, MA, USA) at 100 V for 3 h at 4 °C. Then, 1X TBST is used to wash the membrane at least three times, then the membrane was kept for 2 h in 5% skimmed milk dissolved in TBST (0.1% Tween 20, 150 mM NaCl, and 25 mM Tris, pH 7.5), followed by overnight incubation with host rabbit IL-4 (1:2000 dilution in TBST with 5% BSA, Invitrogen #cat: PA 5-115416, Lot# WK3439657). The next day, the membrane was washed with 1X TBST repeatedly, three times, followed by incubation in a secondary Goat anti-rabbit IgG coupled to horseradish peroxidase for three hours (1:5000 dilution in TBST, Cell Signaling, Invitrogen). Primary antibodies used were caspase antibody (Cat# 31A1067, dilution 1:7500) and β-actin antibody (DSHB, dilution 1:7500). The secondary antibodies utilized were sourced from DSHB, including Goat anti-mouse IgG (Cat# 0600680251730) and Goat anti-rabbit IgG (Cat# 0600580051730). The blots were incubated with Goat anti-Rabbit or Goat anti-mouse IgG antibodies conjugated to horseradish peroxidase and diluted to a concentration of 1:10,000. Target proteins were detected using an ECL kit (Bio-Rad), and the image was captured using a ChemiDocTMXRS + imager (Bio-Rad).

### 2.16. Analysis of Histological Parameters Using H&E and DAPI Staining

The experimental animals’ tissues were quickly removed, washed with saline, blotted, and weighed. Histological staining of liver samples was performed using Hematoxylin and Eosin (H&E) dye. The liver was fixed in Bouin’s fixative overnight, followed by washing under flowing water, and graded ethanol (30, 50, 70, 90, and 100%) was used for the dehydration. After the dehydration, the organs were embedded in a wax block. The fixed tissue was sectioned 10 µm serially using a microtome. The albumin-coated slides were used to place the tissue section and stretched, followed by H&E staining. The liver slide sections were mounted and evaluated under the light microscope.

The DAPI staining of liver specimens was used to detect apoptosis-associated morphological changes in the samples. The fixed samples are washed with 1X PBST (PBS containing 0.1% Tween), followed by DAPI (1 µg L^−1^) staining of the samples for 10 min at 4 °C in the dark. The mounting was performed by DABCO and observed with a fluorescence microscope.

### 2.17. Analysis of Immunohistochemical Properties

To examine the expression of IL-4 in liver tissue, immunostaining was performed. The rat’s liver was fixed in 4% formaldehyde, then paraffin blocks were prepared and, simultaneously, the liver section was cut into 15 µm sections using a cryotome. The sections were attached to positively charged poly-L-lysine glass slides. Paraffin-embedded sections were deparaffinized and hydrated in pure alcohols. Then, the slides were washed twice with 1X PBST for 5 min each. After washing, the slides were subjected to IL-4 primary antibody, host rabbit (Invitrogen #cat: PA 5-115416, Lot# WK3439657), 1:5000 dilution, overnight at 4 °C. The slides are then washed with 1X PBST three times, 10 min each, and then the slides are incubated with the secondary antibody Goat anti-rabbit IgG (1:500 dilutions, Cat#A11008, Lot# 2382186 Invitrogen) for 3 h at RT. After incubation, the sections were washed three times with 1X PBST for 10 min each, and then mounted in DABCO (product no. 157609, M.P. Biomedical). Images were taken using a Nikon Eclipse Ni fluorescence microscope (Nikon, Tokyo, Japan).

### 2.18. In Silico Study on Hepatotoxicity of the Liver with CORT and Possible Protection by GA

Molecular docking was performed using the AutoDock Vina 1.1.2. The structures of the selected ligands were generated using ChemDraw 16.0, and energy minimization was carried out with Chem3D 16.0. The crystal structures of Keap1 (PDB ID: 4ZY3), IKKβ (PDB ID: 4KIK), and COX-1 (PDB ID: 4O1Z) were retrieved from the Protein Data Bank. The protocol was validated using AutoDock Vina 1.1.2 and Pymol. During the preparation of protein and ligand, all heteroatoms and water molecules were removed, followed by the addition of polar hydrogen atoms and Gasteiger charges to the structures. A grid box of 40 × 40 × 40 points with a spacing of 0.375 Å was constructed around the active site of each protein based on the binding site of the co-crystallized ligands and the re-docked ligand. The grid center co-ordinates were set at x = −50.628, y = −2.989, z = −6.081 for Keap1 (bardoxolone methyl binding pocket), x = 50.313, y = 28.268, z = −58.112 for IKKβ (auranofin binding pocket), and x = 241.305, y = 102.081, z = 23.746 for COX-1 (diclofenac binding pocket). Visualization and analysis of protein–ligand interactions, including hydrogen bonding, hydrophobic contacts, and π–π interactions, were carried out using the ligand interactions module of BIOVIA Discovery Studio Visualizer 2021 (version 19.1.0.18287) [47].

### 2.19. Statistical Investigation

The one-way ANOVA test, followed by Tukey’s post hoc test, with a significance level of *p* < 0.05, was utilized for the statistical analysis. The mean ± SD values of the analyzed data were compared to control or CORT-treated rats at *p* < 0.05. GraphPad Prism 5.0 software was employed for the statistical analysis.

## 3. Results

### 3.1. Impact of CORT and GA on the Physical and Behavioral Parameters of Experimental Rats

**Figure 1:**
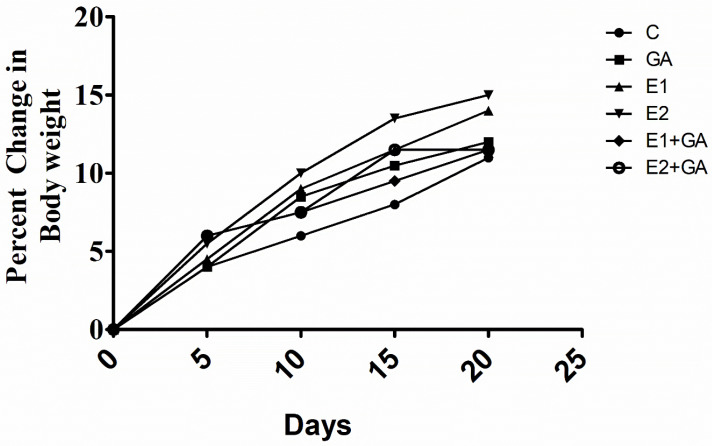
Effect of treatment of the CORT and GA on body weight. C = control, GA = gallic acid (50 mg kg^−1)^, E1 = CORT (15 mg kg^−1^), and E2 = CORT (30 mg kg^−1^), respectively. GA + E1 and GA + E2 represent the pretreatment of rats with GA followed by CORT, respectively.

The body weight of rats was taken on the starting day of the experiment and ranged from 100 to 150 g. An average increase in body weight of 10% was noted in the control group animals after 21 days. It was observed that CORT treatment caused a 15% increase in the body weight of animals. However, no significant change was observed in the group pre-treated with GA followed by CORT (Figure 1, Table 1).

The impact of CORT on the behavioral parameters in the rats, such as locomotion, water intake, food intake, thigmotactic response, rest and sleep, hyperactivity, aggression, biting, rubbing, and alertness, was also observed after the treatment of animals with GA, CORT, and their combinations. The results recorded are shown in Table 1. The locomotion, thigmotactic response, and alertness significantly decreased in the experimental animals treated with CORT compared to the control. However, water and food intake, rest and sleep, and rubbing patterns were slightly increased due to CORT treatment. However, almost no hyperactivity and aggression were observed in the animals by exposure to CORT (Table 1).

### 3.2. Evaluation of CORT-Induced Oxidative Stress in Rats and the Protective Effect of GA

#### 3.2.1. Impact of CORT on the Levels of Protein and Nonenzymatic Antioxidants

The concentration of protein in the liver homogenates of rats in different experimental groups was determined, and the results are shown in Table 2. The protein content in the liver of rats from all experimental groups was found to be approximately 70 mg/g liver. The data suggested no significant change in the hepatic protein content of the rats with GA- and CORT-treated groups compared to the control (Table 2).

The nonenzymatic antioxidant levels, such as GSH, MDA, and PCO, were determined in the liver of the rats from different experimental groups, as described in Materials and Methods. The findings outlined in Table 2 revealed a significant reduction in hepatic glutathione levels in rats administered with CORT, ranging from 41.4% to 52.1% with increasing doses from 15 mg kg^−1^ to 30 mg kg^−1^, respectively, compared to the control group. Conversely, in the animal cohort pre-treated with GA before CORT administration, there was a notable improvement in GSH levels, exhibiting statistical significance (*p* < 0.05) when juxtaposed with the CORT-treated group. Notably, administration of GA did not yield any discernible impact on liver GSH levels.

Lipid peroxidation was assessed through MDA levels in liver tissue homogenates. The findings, outlined in Table 2, demonstrated that the MDA levels were nearly identical in the control and GA groups. Nonetheless, the oral administration of CORT resulted in a noteworthy (*p* < 0.05) elevation in MDA content by 28% in the liver compared to the control group. Rats that received pretreatment with GA before CORT exhibited substantially reduced MDA levels when contrasted with CORT-treated rats. The MDA levels in rats pre-treated with GA followed by CORT closely resembled those of normal rats (Table 2). Furthermore, the impact of CORT on the concentration of protein carbonyl (PCO) in the hepatic tissues was evaluated. The outcomes revealed a significantly (*p* < 0.05) higher generation (34%) of PCO in liver tissues exposed to CORT as opposed to the control group. Conversely, when rats underwent pretreatment with GA followed by CORT, the PCO levels in liver tissues showed no significant difference compared to the control group (Table 2).

#### 3.2.2. Impact of CORT and GA on the Activities of Antioxidative Enzymes in Rat Liver

**Figure 2:**
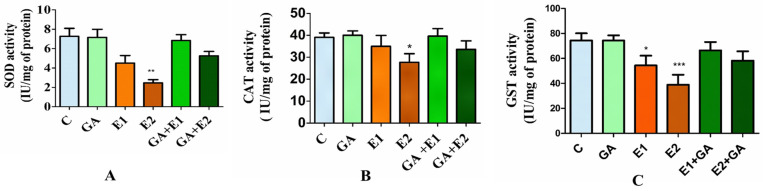
The impact of CORT and GA treatment on the enzymatic activities of Superoxide Dismutase (SOD; (**A**)), Catalase (CAT; (**B**)), and Glutathione S-Transferase (GST; (**C**)) in the hepatic homogenates of rodents was examined. The findings were presented as the mean ± standard deviation (*n* = 6), where *, **, and *** represent statistical *p*-value significance at levels of <0.05, <0.01, and <0.001, respectively. C = control, GA gallic acid 50 mg kg^−1^, E1 = CORT (15 mg kg^−1^), and E2 = CORT (30 mg kg^1^), respectively. GA + E1 and GA + E2 represent the pretreatment of the animals with GA followed by CORT.

The impact of CORT treatment and the protective effect of GA on the enzymatic antioxidant activities, such as SOD, CAT, and GST, were evaluated in the hepatic tissues of rats exposed for 21 days. The results depicted in Figure 2 showed that the CORT markedly influenced the antioxidative activities of enzymes. The SOD activity was significantly decreased (48%) due to the treatment with CORT (30 mg kg^−1^). There was a slight increase (15%) in the activity of SOD, denoted by treatment with GA (50 mg kg^−1^) alone. However, the pretreatment of rats with GA followed by CORT caused significant recovery in the SOD activity (58%) as compared to the CORT-treated group (Figure 2A). Similarly, the activity of CAT was found to be significantly reduced by 34% due to CORT treatment. However, the pretreatment of GA followed by CORT caused significant recovery in the activity of CAT near the values of control (Figure 2B). The impact of CORT on the GST activity was also evaluated in the liver of rats. The results presented in Figure 2C showed that the activity of GST decreased significantly by 51% compared to the control. However, the pretreatment of GA followed by CORT caused a significant (*p* < 0.05) improvement in the GST level. The findings of our investigation illustrated that the administration of 30 mg kg^−1^ of CORT leads to a notable reduction in antioxidant status through the inhibition of SOD, CAT, and GST activities. The prior exposure of rats to GA improved the cellular redox system in rats subjected to CORT.

### 3.3. Impact of CORT and GA on the Oxidative Stress Index (OSI) of the Rat Liver

The OSI value was calculated for the liver of rats after the 21-day treatment with CORT and GA. The OSI values have been presented in Table 3. The OSI values (70–122%) were higher in the liver of CORT-treated rats than in the control. The prior application of GA followed by CORT treatment indicated the OSI values near the control.

### 3.4. Effect of CORT and GA on the Serum Cortisol Level and Serum Glucose

**Figure 3:**
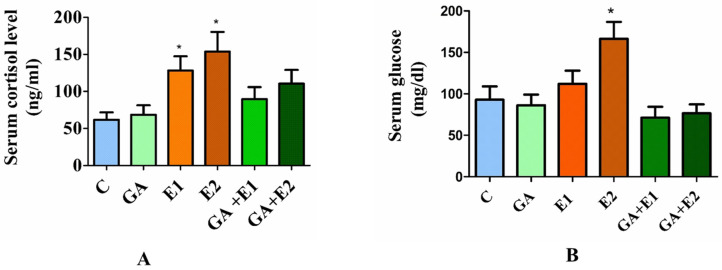
Effect of CORT and GA treatment on the Serum cortisol (**A**) and Serum glucose (**B**). The results are presented as the mean ± SD (*n* = 6), where * indicates a *p*-value significance of <0.05 compared to the control, as determined by the Tukey test. C = control, GA = gallic acid (50 mg kg^−1)^, E1 = CORT (15 mg kg^−1^), and E2 = CORT (30 mg kg^−1^), respectively. GA + E1 and GA + E2 represent the pretreatment of rats with GA followed by CORT, respectively.

The rats treated with CORT were found to have a significant increase in cortisol levels (3-fold) and serum glucose (1.5-fold) compared to the control group. However, the pretreatment with GA followed by CORT showed a decline in cortisol level compared to the untreated one (Figure 3A), and the serum glucose of rats was near normal (Figure 3B).

### 3.5. Impact of CORT and GA on the ATP Level in the Rat Liver

**Figure 4:**
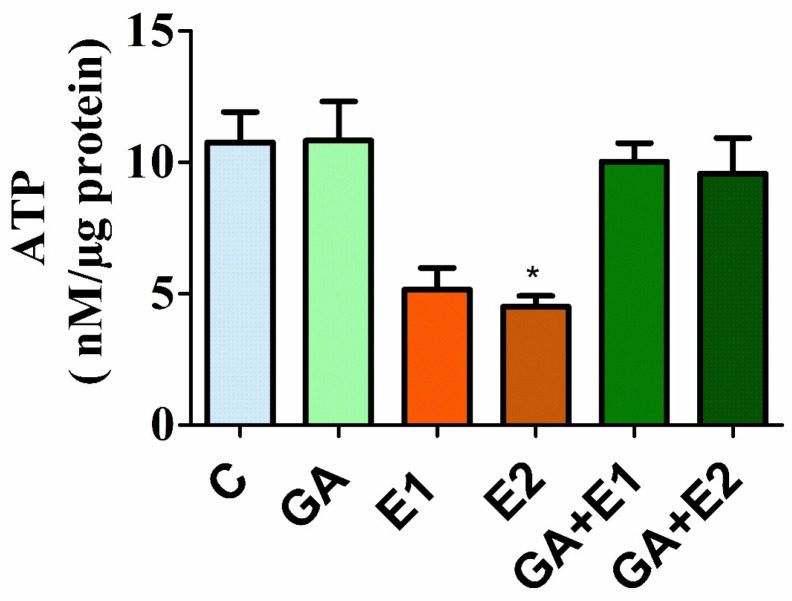
Impact of CORT and GA treatment on the level of ATP. The results are represented as the mean ± SD (*n* = 6), where * indicates p values are significant at <0.05 using the Tukey test. C = control, GA gallic acid 50 mg kg^−1^; E1 = CORT (15 mg kg^−1^), and E2 = CORT (30 mg kg^−1^), respectively; GA+ and GA + E2 represent the group of animals pre-treated with GA followed by CORT.

The impact of CORT on the level of ATP was assessed in the homogenate of rat liver, as described in Materials and Methods. The results shown in Figure 4 demonstrated a significant (*p* < 0.05) decrease (50–55%) in the level of ATP in the rat liver due to CORT treatment. The group of animals pre-treated with GA followed by CORT exhibited significant recovery in the ATP level; the values were close to the control.

### 3.6. Effect of CORT on the Levels of Serum ALT and AST and the Ameliorative Effect of GA

**Figure 5:**
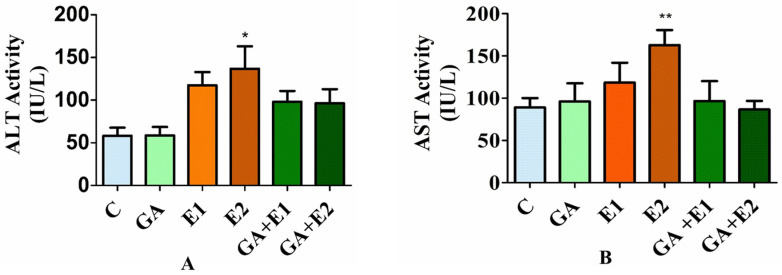
Impact of CORT and GA on the enzymatic activities of ALT (**A**) and AST (**B**) in the rat’s serum. The findings were presented as the mean ± standard deviation (*n* = 6), where * and ** represent statistical significance at *p* < 0.05 and *p* < 0.01, respectively. C = control, GA (50 mg kg^−1^), E1 = CORT (15 mg kg^−1^), and E2 = CORT (30 mg kg^−1^), respectively; GA + E1 and GA + E2 indicate the pretreatment of animals with GA followed by CORT.

The impact of CORT and GA on the levels of hepatic function enzymes, i.e., the transaminases (ALT and AST) in rat blood serum, was evaluated using the protocol described in the materials and methods section. The results indicated in Figure 5A show a 55–60% increment in the level of ALT and 40–48% in the activity of AST in the CORT-treated group of animals compared to the control (Figure 5B). However, the pretreatment of animals with GA followed by CORT reflected a substantial recovery in the levels of these enzymes near to that of control.

### 3.7. Impact of CORT and GA on the Activities of LDH, ALP, and ACP in Rat Blood Serum

**Figure 6:**
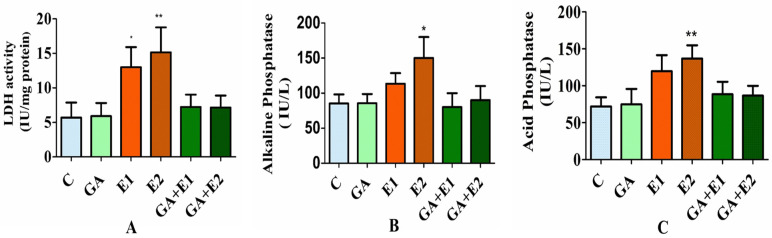
Impact of CORT and GA administration on the activities of LDH (**A**), ALP (**B**), and ACP (**C**) in the rat’s serum. The results are expressed as the mean ± SD (*n* = 6); * and ** indicate the *p* values significant at <0.05 and <0.01, respectively. C = control, GA (50 mg kg^−1^); E1 = CORT (15 mg kg^−1^), and E2 = CORT (30 mg kg^−1^), respectively. GA + E1 and GA + E2 indicate the pretreatment of animals with GA followed by CORT.

The evaluation of the impact of CORT and GA on the activities of LDH and phosphatases in the blood serum of rats was made using the procedure described in Materials and Methods. The results presented in Figure 6 suggested that there was approximately a three-fold increase in LDH activity (Panel A) due to the treatment of animals with CORT compared to the control. Similar observations were recorded with the response of phosphatase activities (ACP and ALP) to the CORT treatment. The exposure of CORT resulted in a 25–43% increment in the ALP activity (Panel B) and about a 50% rise in the ACP activity (Panel C). However, the prior application of GA followed by CORT caused the reversal of the CORT-induced impact on these enzymes’ activities; the values appeared close to the normal group of animals. The group of animals treated with GA only showed a response similar to that of the control (Figure 6A–C).

### 3.8. Western Analysis of the Expression Levels of Procaspase-3 and Interleukin-4 (IL-4) in the Animals Treated with CORT and GA

**Figure 7:**
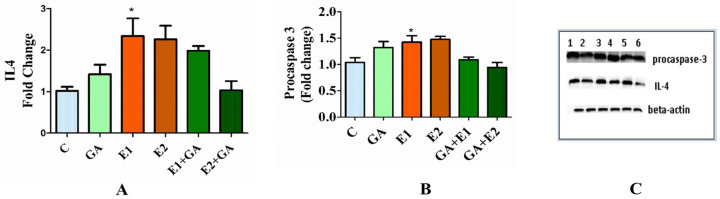
Effect of CORT and GA treatment on the expression of IL-4 (Panel (**A**)), procaspase-3 (Panel (**B**)), and expression in the liver homogenates of rats. The results are expressed as the mean fold change (*n* = 6). Representative Western blot for IL-4 (31 kDa), procaspase-3 (14 kDa), and β-actin (42 kDa) are shown in Panel (**C**); 1—control (C), 2—GA (50 mg kg^−1^), 3—E1 (15 mg kg^−1^), and 4—E2 (30 mg kg^−1^) CORT, respectively. 5—GA + E1 and 6—GA + E2 indicate the pretreatment of animals with GA followed by CORT exposure. * Indicates the *p*-values are significant at <0.05.

To detect the influence of CORT and GA on the expression of chemokine IL-4 and procaspase-3 in the hepatic tissues, their expression analysis was monitored by Western blot analysis using the procedure as described in Materials and Methods. The results presented in Figure 7A illustrated a significant rise (two-fold) in the level of IL-4 (31 kDa) and about a 40% increment in procaspase-3 (14 kDa) expression (Figure 7B) compared to the control. Nevertheless, the prior treatment of the experimental animals with GA followed by CORT indicated that the expression level values of IL-4 and procaspase-3 were close to the control (Figure 7C).

### 3.9. Immunohistochemical Analysis of the Impact of CORT and GA on IL-4

**Figure 8:**
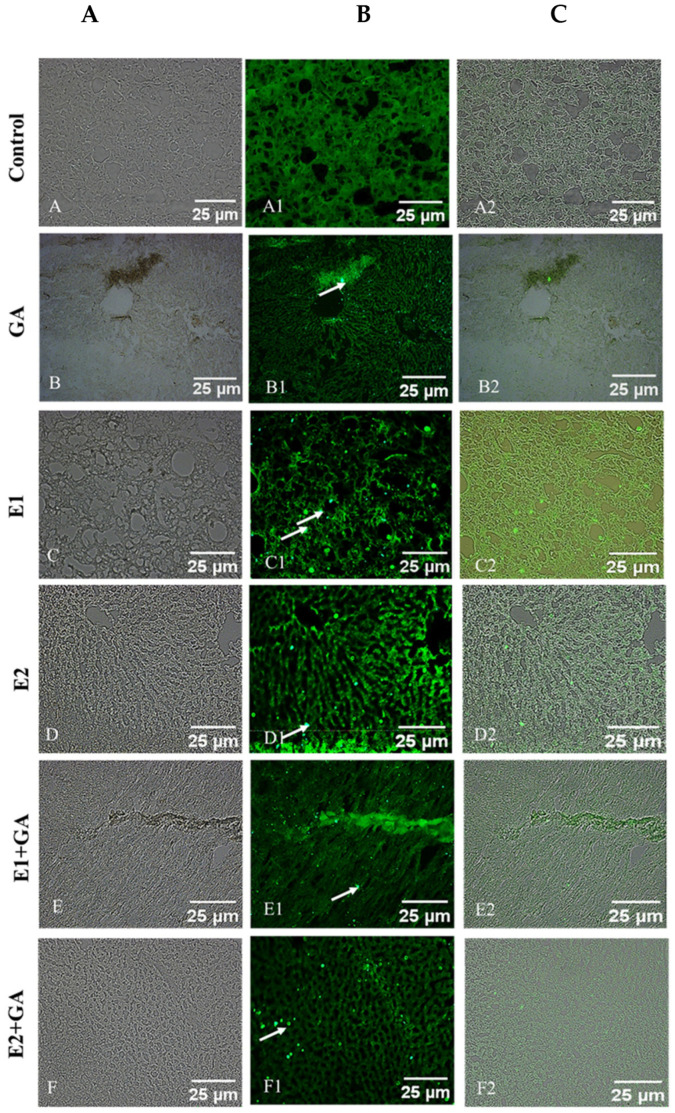
Immunohistochemical analysis of IL-4 expression in liver specimens with CORT and GA treatment for 21 days. *n* = 6, GA (50 mg kg^−1^), E1 = CORT (15 mg kg^−1^), E2 = CORT (30 mg kg^−1^), GA + E1 and GA + E2 indicate the pretreatment of GA followed by exposure to E1 and E2. The arrow indicates the impact of CORT on the expression of IL-4 in rat liver tissues. Panel (**A**) (**A**–**F**) =Bright Field, middle Panel (**B**) (**A1**–**F1**) = Green Field, left side is Panel (**C**) (**A2**–**F2**) = Merged (Bright Field Green Field).

As indicated in Figure 8, CORT had significantly impacted the expression of IL-4. This effect was validated by immunohistochemical analysis of hepatic tissues. The results are shown in Figure 8. In the normal state, IL-4 was found to be mainly concentrated in the area surrounding the central vein and portal region, indicating the basal level of expression of IL-4 in the portal area (Figure 8). The expression level of IL-4 in the CORT-treated group was found to be significantly increased compared to the control, as indicated by immunohistochemical staining (E1 and E2). However, the pretreatment of animals with GA followed by CORT displayed significant amelioration in this context, with the values reaching close to normal. (Figure 8) A qualitative assessment of the immunohistochemical analysis of the impact of CORT on IL-4, as shown in Figure 8, has been summarized in Table 4.

Summary of immunohistochemical expression of IL-4: GA = GA, 50 mg kg^−1^, E1 = 15 mg kg^−1^, and E2 = 30 mg kg^−1^ CORT exposure group, E1 + GA, and E2 + GA indicate the pretreatment of animals with GA followed by CORT. Each liver slide was examined under a fluorescence microscope, and the intensity of the changes was scored using the scale of basal (-); low (+); modest (++), and higher (+++).

### 3.10. Impact of CORT and the Ameliorative Effect of GA on the Histological Status of Rat Liver as Observed by DAPI and H&E Staining

**Figure 9:**
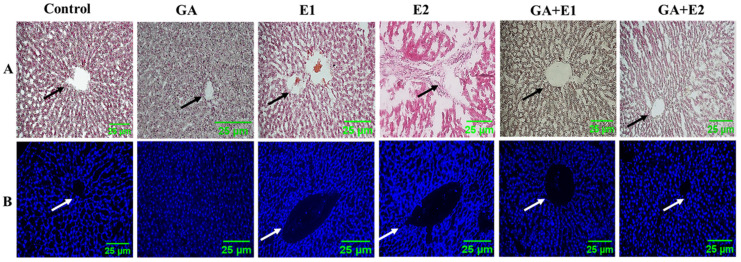
Histological alteration in the liver with CORT and the GA treatments in rats with corticosterone and GA treatment for 21 days. *n* = 6, control = control group, GA (50 mg kg^−1^), E1 (15 mg kg^−1^), and E2 = (30 mg kg^−1^) corticosterone exposure group, GA + E1 and GA + E2 indicate the pretreatment of animals with GA followed by CORT exposure. The black arrow in H&E staining and the white arrows represent the changes in the central vein. (**A**) H&E staining and (**B**) DAPI staining. The arrows indicate the hepatic region changing due to CORT exposure.

To assess the effect of CORT and the protective effect of GA on the liver tissues of rats, the tissues were taken for histological examination using H&E and DAPI staining, as shown in Materials and Methods. The microscopic examination of liver tissue slides showed acini and hexagonal lobules are the characteristics of liver histological architecture (Figure 9). There was a central vein in the middle of the hexagonal lobules. It was observed that the treatment of rats with CORT resulted in alterations in the liver tissue architecture. Due to CORT administration, the hepatic tissues exhibited necrosis and infiltration of cells, granular cytoplasm, and dilation in the sinusoids with vacuolation, indicating damage to the liver. The data reflected the disorganization of the central vein and hepatic cords upon the treatment with 15 mg/kg and 30 mg/kg doses of CORT. The effect observed was dose-dependent. The pretreatment of animals with GA followed by CORT showed significant recovery from the CORT-induced hepatic damage. The results are summarized in Table 5.

Table 5: Summary of the impact of CORT and GA on the architectural parameters of rat liver, as shown in the Figure. 9, GA = GA, 50 mg kg^−1^, E1 = CORT, 15 mg kg^−1^, and E2 = CORT, 30 mg kg^−1^), GA + E1 and GA + E2 indicate the pretreatment of animals with GA followed by CORT; CNH: coagulative necrosis in hepatocytes, CCv: congestion in central vein; DH: degenerations of hepatocytes; H: degree of hemorrhage; DHt: degeneration in the hepatic triad, ND: nuclear disruption, VD: vacuolar degeneration; each liver specimen was meticulously analyzed, and the extent of the alterations was assessed based on a grading system ranging from absent (-) to severe (+++) [42].

### 3.11. In Silico Molecular Docking Analysis of GA with Keap1, IKKβ, and COX-1

To explore the molecular mechanisms underlying the hepatoprotective, antioxidant, and anti-inflammatory properties of GA, molecular docking studies were conducted against three key regulatory proteins, Kelch-like ECH-associated protein 1 (Keap1), IκB kinase β (IKKβ), and cyclooxygenase-1 (COX-1).

**Figure 10:**
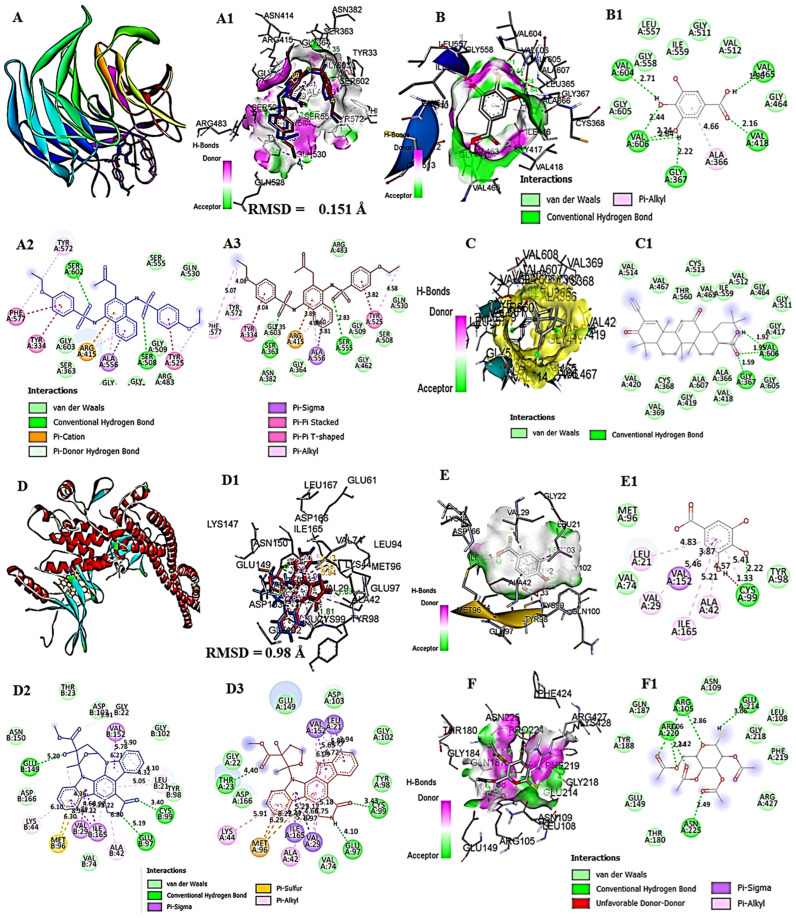

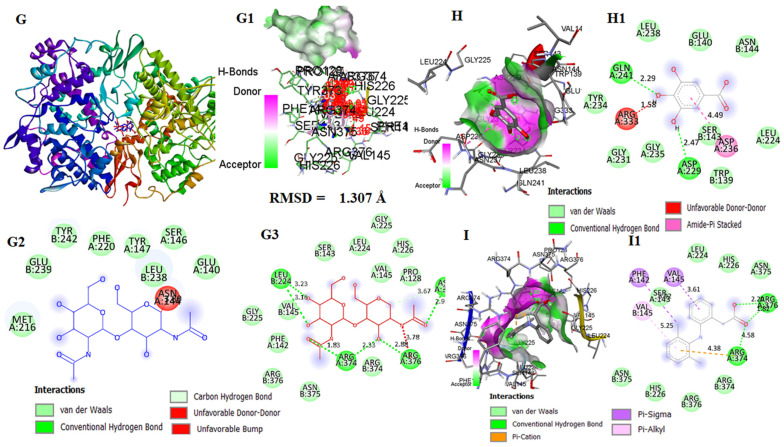
Molecular docking interactions of GA and reference compounds with target proteins. (**A**,**A1**,**D**,**D1**,**G**,**G1**) showed the comparison of binding mode for co-crystallized (blue) and re-docked ligand (red) shown stick representation, and the root mean square deviation (RMSD) indicted the ligand has bound to the protein backbone 0.151 Å, 0.98 Å, 1.307 Å, the amino acid residues interaction (**A2**,**D2**,**G2**) co-crystallized (blue) and (**A3**,**D3**,**G3**) re-docked ligand (red), (**B**,**B1**) showed the GA–Keap1 complex (binding energy: −6.9 kcal/mol); (**C**,**C1**) reference compound bardoxolone methyl–Keap1 complex (−9.0 kcal/mol); (**E**,**E1**) GA–IKKβ complex (−6.0 kcal/mol); (**F**,**F1**) reference compound auranofin–IKKβ complex (−5.1 kcal/mol); (**H**,**H1**) GA–COX-1 complex (−6.2 kcal/mol) and (**I**,**I1**)) reference compound diclofenac–COX-1 complex (−7.1 kcal/mol). Docking was performed using AutoDock vina 1.1.2. Hydrogen bonding and hydrophobic interactions between GA and key active-site residues are indicated.

Comparative binding analyses between the co-crystallized and re-docked ligands revealed highly conserved conformations within the Keap1, IKK, and COX-1 active sites, with RMSD values of 0.151 Å, 0.98 Å, and 1.307 Å, respectively, confirming the accuracy and reliability of the docking approach. The co-crystallized and re-docked poses exhibited consistent key interactions through hydrogen bonding and hydrophobic contacts with critical residues—ARG415, SER555, GLN530, and TYR572 in Keap1; ARG452, GLN451, MET455, and GLN611 in IKK; and SER143, ASP229, GLN241, and TYR333 in COX-1 supported the structural stability of the ligand–protein complexes within their respective binding domains (Figure 10A–A3,D–D3,G–G3).

Molecular docking of GA with Keap1 revealed a favorable binding conformation within the Kelch domain, with a binding energy (ΔG) of −6.9 kcal/mol, suggesting a stable interaction profile. GA was well accommodated within the active cavity, forming hydrogen bonds with GLY367 (2.60 Å), ALA366 (3.64 Å), and GLY605 (4.49 Å), along with alkyl interactions involving VAL418 (5.38 Å) and VAL465 (4.71 Å). Additional van der Waals contacts with GLY417, GLY511, VAL604, and GLY606 further stabilized the complex. The 2D interaction map confirmed these interactions, with a minor, unfavorable donor–donor contact at ILE559 (5.23 Å) (Figure 10B,B1).

Similarly, bardoxolone methyl–Keap1 (reference compound) exhibited a stronger binding affinity (ΔG = −9.0 kcal/mol), with the ligand stably occupying the hydrophobic cavity of Keap1. The interaction network involved residues VAL369, CYS368, GLU367, LEU365, ALA366, VAL418, VAL465, LEU468, ASN469, ARG470, VAL512, CYS513, VAL514, THR560, VAL561, ALA606, and ALA607. Hydrogen bonds were observed with ASN469 (2.42 Å), VAL369 (2.31 Å), and ALA607 (3.55 Å). Meanwhile, hydrophobic and π-alkyl interactions were observed with VAL467, VAL465, and LEU468, while van der Waals contacts involved CYS368, GLY419, VAL420, and GLY511, contributing to complex stabilization. The 2D interaction profile corroborated these findings, showing a consistent hydrogen-bonding network supported by extensive hydrophobic interactions (Figure 10C,C1).

Docking analysis of GA with IKK revealed a binding energy (ΔG) of −6.0 kcal/mol, indicating a stable and favorable interaction. GA was well accommodated within the IKK active-site cavity and stabilized through a combination of hydrogen bonding, van der Waals forces, and π–sulfur interactions. The key interacting residues included ARG452, GLN448, GLN451, MET455, MET456, LEU459, LEU547, GLN548, SER550, GLN543, and GLN611. Hydrogen bonds were formed with GLN451 (5.05 Å), LEU547 (4.26 Å), and GLN611 (5.05 Å), while a π–sulfur interaction with MET455 (6.21 Å) further contributed to ligand stability. Additional van der Waals interactions with ARG452, GLN448, and LEU459 reinforced the overall binding affinity. The 2D interaction profile confirmed these observations, highlighting the same hydrogen bonding and π–sulfur contacts, along with extensive van der Waals stabilization (Figure 10E,E1).

In contrast, docking of auranofin (reference compound) with IKK exhibited a binding energy of −5.1 kcal/mol, representing the best-ranked pose among the reference conformations. Auranofin occupied the IKK binding pocket, forming hydrogen bonds with ASN462 (2.24, 1.99 Å), GLN541 (2.92 Å), VAL545 (2.92 Å), and ARG549 (5.20, 5.86 Å). The interaction surface displayed well-defined donor and acceptor regions corresponding to these polar interactions. Additional van der Waals contacts with THR542, GLN548, LEU459, ILE544, and VAL545 further contributed to complex stabilization (Figure 10F,F1).

The docking of GA into the COX-1 active site (Figure 10H,H1) exhibited a binding affinity of −6.2 kcal/mol, indicating a stable and favorable interaction. GA was anchored through several conventional hydrogen bonds with residues THR206 (3.18 Å), TYR385 (2.81 Å), HIS207 (2.04 Å), and HIS388 (2.44 Å), suggesting strong polar interactions. Additional van der Waals contacts were identified with TRP387, ALA199, THR206, and LEU390, enhancing the binding stability. The ligand also engaged in π–π stacking and π–cation interactions, contributing to aromatic stabilization. Although a minor unfavorable donor–donor contact was observed between ALA202 and GLN203 (4.98 Å), it did not affect the overall binding conformation of the COX-1–GA complex.

In comparison, diclofenac docking within the COX-1 catalytic site (Figure 10I,I1) yielded a binding affinity of −7.1 kcal/mol, slightly higher than GA. The 3D and 2D interaction maps revealed that diclofenac was deeply embedded in the enzyme pocket, forming key van der Waals interactions with SER353, GLY526, LEU359, and MET522. Crucial π–sigma and π–alkyl interactions were identified with TYR355 (5.08 Å), ALA348 (3.49 Å), and VAL344, contributing to hydrophobic stabilization. Additionally, ARG120, TRP387, and PHE518 were involved in π-interactions, supporting ligand orientation and retention within the COX-1 active site.

## 4. Discussion

The steroidal drugs are widely prescribed by physicians as a drug of choice to treat various chronic inflammatory conditions such as rheumatoid arthritis, asthma, intestinal bowel diseases, allergic conditions, etc. [7]. Studies on the treatment of diseases using CORT have reported the onset of several side effects in patients, namely osteoporosis, weight gain, hyperglycemia, mood elevation, etc. [60]. Recent reports have indicated that the application of some plant-based bioactive molecules with steroidal drugs was able to reduce CORT-induced toxicity. However, not much information is available in this context.

The present study has investigated the CORT-induced alterations in the morphological, physiological, and biochemical indices in rats exposed to sub-acute concentrations of 15 and 30 mg kg^−1^ body weight for 21 days, and the protective effect of GA (50 mg/kg^−1^). In this study, the treatment of rats with CORT caused a marginal increment (15%) in body weight compared to the control. Similar reports have been presented by Wang et al. [8]. In contrast, a further study indicated a significant decrease in the body weight of rats exposed to CORT (10, 20, 40 mg kg^−1^) for 21 days [25]. The observations recorded in the present study indicated an increase in body weight only after two weeks of treatment with CORT. However, the pretreatment of GA followed by CORT treatment restricted the increase in body weight, indicating its protective effect; the recorded observations showed agreement with previous studies [61].

High blood glucose levels are the main features of the diabetic condition [62,63]. According to the American Diabetes Association (2017), the application of glucocorticoids leads to abnormally high blood glucose levels in patients with or without a diabetic history, resulting in the development of steroid-induced diabetes mellitus [64]. The results from the present study revealed a significant rise in the level of blood glucose and body weight in the CORT-treated rats, similar to the observations recorded by other workers [8,65,66].

Glucocorticoids additionally facilitate the hepatic synthesis of ceramide, a sphingolipid, which is significantly correlated with hepatic insulin resistance [9,65]. GA was observed to mitigate elevated blood glucose concentrations in diabetic contexts [43]. The liver serves as the primary organ to synthesize nutrients and hormones essential to glucose homeostasis. The liver augments gluconeogenesis to preserve euglycemia in insufficient blood glucose. Under stress, glucocorticoids elevate glucose concentrations in the liver, ensuring the organism possesses adequate glucose to sustain cerebral function and overall survival. In response to heightened glucocorticoid concentrations, the liver activates enzymes that are pivotal in the biosynthesis of glucose [16].

The use of CORT in different clinical conditions has been shown to induce oxidative stress in different body organs [7,67,68]. Oxidative stress has been identified as a critical contributor to hepatic toxicity. In the current study, the concentrations of nonenzymatic oxidative indicators such as malondialdehyde (MDA), protein carbonyls (PCO), and glutathione (GSH) exhibited significant alterations as a result of CORT administration; specifically, the levels of MDA and PCO demonstrated an increase, whereas GSH levels experienced a decline. These findings are consistent with those reported by other researchers [10,25]. The elevated MDA concentration serves as an indicator of the extent of lipid peroxidation and oxidative stress. The results of the present study indicated about a 40% increase in MDA level in the rat liver treated with CORT (15 and 30 mg kg^−1^ body weight), similar to the findings of Fouad et al. and other researchers, who showed an increase in the MDA concentration to about three-fold in the blood serum and the renal tissues, respectively, after the treatment with CORT [22]. Another nonenzymatic antioxidant, i.e., GSH, was found to have decreased in the rat liver by 42% due to CORT treatment. Zafir and Banu have reported a significant reduction in GSH levels in the brain, liver, and heart of the CORT (40 mg kg^−1^)-treated animals [22]. Similarly, Fouad et al. showed about a 20% reduction in the GSH level in the renal tissues due to methylprednisolone (MPL, 100 mg) treatment [27]. These results indicate that exposure of animals to CORT might be adversely influencing the synthesis of GSH in the tissues, as there was a significant rise in the lipid peroxidation and production of free radical species in the rat liver. The reduced form of GSH interacts with free radicals, preferably oxygen-free radicals or lipid peroxides, and produces fatty acids and water.

Earlier reports have suggested that the application of steroidal drugs may cause perturbations in the levels of enzymatic activities such as CAT, SOD, and GST [8,25]. The role of SOD in removing excess superoxide ion free radicals is by converting them into hydrogen peroxide, and CAT in diminishing hydrogen peroxide into oxygen and water, and preventing the hydroxyl radicals. The GST catalyzes the biotransformation of xenobiotics via a conjugation reaction. The data from the present study indicated a significant loss of these enzymatic antioxidant activities in rat liver exposed to CORT (15 and 30 mg kg^−1^) for 21 days. The levels of reduction in the activities of SOD, CAT, and GST in the rat liver were 48%, 34%, and 51%, respectively, due to treatment with the maximum concentration of CORT (30 mg kg^−1^), the effect being concentration-dependent. Zafir and Banu have reported a decrease in the activities of SOD, CAT, and GST by a tune of 36%, 60%, and 63%, respectively, due to the treatment of rats with CORT (40 mg kg^−1^ body weight) [25]. According to Faud et al., there was a 50% reduction in the activity of SOD due to the treatment of MPL (100 mg) [27]. In another study by Lew et al. on PC12 cells, there was a 30% decrease in the activity of SOD and a 20% decline in the CAT activity due to CORT treatment [69]. The pretreatment of animals with GA caused a significant level of protection from the toxic effects of CORT. Esmaeilzadeh et al. have shown mitigation of diclofenac-induced liver toxicity by GA via modulation of the antioxidant defense system and decline in IL-1β gene expression in rats [61].

In the present study, the treatment of CORT resulted in a significant decrease in the level of hepatic ATP concentration, which was substantially normalized by pretreatment with GA. The decreased concentration of ATP might be related to an abnormality in mitochondrial function. A study by Fujita et al. found that corticosterone prevents mitochondria from producing ATP and lowers complex I activity in the blood at physiological levels [70]. Further investigations revealed that corticosterone significantly increased cell death in GT1-7 cells treated with t-BuOOH due to oxidative stress and low intracellular ATP levels [70]. Prior application of GA protects the level of ATP concentration, which might be associated with its high antioxidant potential. Further research is necessary to elucidate the steroid’s (CORT) mechanisms of action, such as impeding energy production in mitochondria [71,72].

Apoptosis is orchestrated by stimulating diverse cysteine group proteases, described as caspases. These caspases typically remain in the inactive state called procaspases and are subsequently triggered by proteolytic degradation and heterodimerization. The programmed cell death initiation involves the caspase cascade, where ‘starter’ caspases (e.g., caspase-8, 9, 12) are first activated, followed by the division and activation of ‘effector’ caspases (e.g., caspase-3, 6, 7). The effector caspases execute the proteolytic processes that lead to cellular degradation and demise.

Studies in cancer have shown that overexpression of procaspases can influence the sensitivity of cells to chemotherapy-induced apoptosis [41]. The researchers have demonstrated that in skeletal muscle, GC can trigger or activate the apoptotic signaling pathway via caspase-3 activation, fragmentation of DNA, and phosphatidylserine translocation [73].

GCs are generally found to suppress the immune system by suppressing several cytokines and interleukins; however, not every cytokine is suppressed. In different conditions, it remains unaltered or is sometimes expressed [74,75]. In another study on the PCOS model of rats, it was found that there was no significant alteration in the expression of pro-inflammatory and inflammatory cytokines. However, despite this, a slight increase in the interleukins, such as IL-4 and IL-10 was found after GC treatment [76]. IL-4 is classified as a type I cytokine, characterized by a four-α-helical bundle structure, exerting diverse pleiotropic effects across various lineages. Initially identified for its significant impact on B and T cells, IL-4 demonstrates its biological activity on a wide array of target cells from the innate and adaptive immune compartments, originating from many distinct cellular reservoirs.

In the current study, the IL-4 expression increased in hepatocytes compared to the control, which might be related to hepatic damage and promoting cancerous tissue; however, prior application of GA protects from these toxic effects. The property of GA in preventing oxidative damage might be due to its antitumor effect by modulating the prooxidant-to-antioxidant ratio. It was also reported that this molecule inhibits ROS-induced carcinogenesis by reducing lipid peroxidation and increasing the activity of antioxidant enzymes such as SOD, CAT, GR, and GPx [77].

In numerous studies, the application of CORT was found to cause hepatic steatosis and infiltration [78]. In the presence of glucocorticoids, hepatic uptake of circulating non-esterified fatty acids (NEFA) is increased, further promoting gluconeogenesis and hepatic steatosis [79]. This is due to the accumulation of lipids on the surface of the liver [8]. A study by Goh et al. in the hepatotoxic conditions, the eosinophils were recruited onto the hepatocytes and secreted IL-4, which further started the regeneration pathway [80]. Therefore, the high IL-4 expression in hepatocytes after CORT treatment can be attributed to the regulatory effects of GC on hepatocytes, influencing the IL-4 signaling pathway.

To further substantiate the hepatoprotective, antioxidant, and anti-inflammatory effects of GA, observed in vivo, in silico molecular docking analyses were conducted targeting key regulatory proteins involved in oxidative stress and inflammation, Keap1, IKKβ, and COX-1.

GA, a naturally occurring polyphenol, has also been shown to protect against hepatotoxicity induced by tert-butyl hydroperoxide through the activation of the Nrf2, ERK, and Keap1 antioxidant pathways [47]. The Keap1-Nrf2/HO-1/NF-κB pathway is a key signaling route; it plays an essential role in cellular antioxidant responses, anti-inflammatory actions, mitigation of mitochondrial damage, regulation of calcium entry, and control of cell death. Nrf2 acts as a crucial transcription factor that helps counteract oxidative stress by promoting the expression of antioxidant proteins, especially the heme oxygenase-1 (HO-1) enzyme [48]. When there is no stress, Keap1 functions as an E3 ubiquitin ligase substrate adaptor that targets Nrf2 for rapid proteasomal degradation, thereby limiting the cytoplasmic concentration of Nrf2. However, in a stressful situation, it is vice versa [48].

To investigate whether GA could directly inhibit the protein–protein interaction between Keap1 and Nrf2, we assessed their ability to bind to Keap1 using AutoDock Vina molecular docking software. GA exhibited a reliable affinity with Keap1 (−6.9 kcal/mol), comparable to that of bardoxolone methyl (−9.0 kcal/mol), a known Nrf2 activator. The C-terminal Kelch domain of Keap1 is considered the binding site for Nrf2 peptides, and several reported inhibitors that directly bind this site disrupt the Nrf2–Keap1 interaction, promoting Nrf2′s nuclear translocation. Therefore, we hypothesized that GA-induced Nrf2 activation might also involve direct binding to the Kelch domain of Keap1. Keap1 acts as an inhibitor of Nrf2 degradation. Our results showed that GA can effectively interact with the Keap1 binding site and dissociate Keap1 from Nrf2, resulting in the translocation of Nrf2 into the nucleus and the activation of several ARE-driven genes [47,48].

Parallelly, IKKβ plays a pivotal role in activating the NF-κB inflammatory pathway. Under resting conditions, NF-κB (p65/p50) is held inactive in the cytoplasm by IκBα. When cells are exposed to inflammatory stimuli or oxidative stress, IKKβ becomes phosphorylated (phospho-IKKβ), leading to the phosphorylation and degradation of IκBα. This releases NF-κB, allowing it to translocate to the nucleus, where it promotes transcription of pro-inflammatory cytokines. More notably, GA displayed higher binding affinity to IKKβ (−6.0 kcal/mol) compared with the reference compound auranofin (−5.1 kcal/mol), indicating the potential for GA to inhibit NF-κB activation and reduce downstream pro-inflammatory signaling [74].

The inhibition of phospho-IKKβ-mediated NF-κB activation by GA, coupled with the concurrent activation of the Keap1–Nrf2/ARE axis, suggests a coordinated regulatory mechanism that attenuates oxidative stress-induced inflammatory signaling in hepatic tissue.

Furthermore, GA showed fine binding affinity to COX-1 (−6.2 kcal/mol) compared to diclofenac (−7.1 kcal/mol), a widely used non-steroidal anti-inflammatory drug, implying GA’s potential to modulate prostaglandin synthesis and attenuate inflammation. These docking interactions revealed favorable steric compatibility and chemical complementarity, confirmed via visualization of hydrogen bonding and hydrophobic contacts in Discovery Studio.

Collectively, these computational findings support the experimental outcomes by proposing conceivable molecular mechanisms through which GA protects against CORT-induced liver damage. Thus, the in silico reinforces the observed biochemical and histopathological improvements and also highlights the therapeutic potential of GA as a multi-target natural compound capable of modulating oxidative and inflammatory pathways.

## 5. Conclusions

The findings of the current investigation demonstrate that prolonged exposure to CORT leads to the stimulation of free radicals, resulting in oxidative stress through a notable decrease in both enzymatic and nonenzymatic antioxidants in the rat liver. The treatment with CORT resulted in disturbances in the levels of crucial liver biomarkers, interleukins, and indicators of apoptosis, such as procaspases.

The study demonstrates that GA exerts significant hepatoprotective, antioxidant, and anti-inflammatory effects against CORT-induced liver toxicity in Wistar rats. GA administration shows a protective effect on oxidative stress by restoring antioxidant enzyme activity, reducing lipid peroxidation, and normalizing liver function enzymes, as well as modulating proapoptotic and inflammatory markers such as procaspase-3 and IL-4. Histological and immunohistochemical analysis further confirmed the protective effect of GA on hepatic architecture. Importantly, the computational studies provided mechanistic insights into the bioactivity of GA. It showed strong binding affinity with key molecular targets involved in oxidative stress and inflammation. These interactions suggest that GA may modulate the Nrf2/Keap1 antioxidant response, NF-κB signaling via IKKβ, and COX-mediated inflammatory pathways through direct molecular binding, supporting its therapeutic potential. Collectively, the combination of in vivo and in silico findings highlights GA as a promising natural candidate for the prevention and management of stress hormone-induced hepatic injury and associated metabolic dysfunctions.

## Figures and Tables

**Table 1:** Assessment of the impact of CORT administration on the physical and behavioral indices in the experimental rat.

S. No.	Parameters	Control			GA			E1			E2			GA + E1			GA + E2		
1.	Days	7	14	21	7	14	21	7	14	21	7	14	21	7	14	21	7	14	21
2.	Weight (g)	160	170	178	150	165	155	175	185	200	175	200	215	160	170	180	150	158	165
3.	Temperature (°C)	98	97	97	98	97	98	98	98	99	98	99	98	97	99	98	97	98	98
4.	Locomotion	+	++	++	+	++	+++	++	+	+	++	+	+	++	+	++	+	+	++
5.	Water intake	+	++	++	+	++	++	+	++	+++	+	++	+++	+	+	++	+	+	++
6.	Thigmotactic Response	+	++	++	+	++	++	+	+	+	+	+	+	+	++	++	+	++	++
7.	Food intake	+	++	++	+	++	++	+	++	+++	+	++	+++	+	++	++	+	++	++
8.	Rest and sleep	+	++	++	+	++	++	+	+	+++	+	+	+++	+	++	++	+	++	++
9.	Hyperactivity	-	-	-	-	-	+	-	-	-	-	-	-	-	-	-	-	-	-
10.	Aggression	-	-	-	-	-	-	-	-	+	-	+	-	-	-	-	-	-	-
11.	Biting	-	-	-	-	-	-	+	+	+	+	+	+	+	+	-	+	+	-
12.	Rubbing	+	+	+	+	+	+	+	+	++	+	+	++	+	+	+	+	+	+
13.	Alertness	+	+	+	+	+	+	-	-	-	-	-	-	-	-	+	-	-	+

GA: gallic acid; E1: CORT (corticosterone 15 mg kg^−1^); E2: CORT (corticosterone 30 mg kg^−1^); GA + E1 and GA + E2 indicate pretreatment of rats with GA followed by exposure to E1 and E2 [‘+’ indicated mild changes, ‘++’ moderate changes, and ‘+++’ severe changes ‘-’ no change].

**Table 2:** Effect of CORT treatment on levels of GSH, MDA, and PCO contents in the rat liver and amelioration by GA.

Groups	C	GA	E1	E2	GA + E1	GA + E2
Protein (mg g^1^ tissue)	70 ± 3.73	72 ± 2.27 ^ns^	75 ± 3.53 ^ns^	75 ± 3.97 ^ns^	74 ± 2.18 ^ns^	74 ± 2.32 ^ns^
GSH (µg mg^−1^ of protein)	1.86 ± 0.15	1.81 ± 0.23	1.09 ± 0.25	0.89 ± 0.32 **	1.76 ± 0.20	1.20 ± 0.39
MDA (*n* mol mg^−1^ protein)	18.25 ± 1.40	17.90 ± 1.90	22.10 ± 2.30	25.01 ± 3.21 *	19.17 ± 2.31	20.10 ± 2.90
PCO (nmol mg^−1^ of protein)	0.45 ± 0.15	0.40 ± 0.14	0.57 ± 0.15	0.79 ± 0.19 *	0.43 ± 0.17	0.51 ± 0.14

The data are presented as the mean ± standard deviation (*n* = 6). The symbols * and ** denote the statistical significance of *p* values at <0.05 and <0.01, respectively. The abbreviation ns corresponds to non-significant findings compared to the control group, utilizing the Tukey test. The variables GSH (µg mg^−1^ of protein), MDA (nmol mg^−1^ of protein), and PCO (nmol mg^−1^ of protein) were assessed. C = control, GA = gallic acid (50 mg kg^−1^), E1 = CORT (15 mg kg^−1^), and E2 = CORT (30 mg kg^−1^), respectively, GA + E1 and GA + E2 are the CORT + pre-treated GA of mentioned concentrations.

**Table 3:** Effect of CORT and GA on the oxidative stress index (OSI) in rat liver.

Parameter	C (×10^−3^)	GA (×10^−3^)	E1 (×10^−3^)	E2 (×10^−3^)	GA + E1 (×10^−3^)	GA + E2 (×10^−3^)
OSI	0.150	0.147	0.255 (+70%)	0.357 (+122%)	0.163 (+8%)	0.200 (+33%)

Note. The OSI was calculated using the ratio of MDA to antioxidant enzymes. Table 1 and Figure 2. The (+) sign shows a % increase in the ratio of the values of P/A. C = control, GA (50 mg kg^−1^), E1 = CORT (15 mg kg^−1^), and E2 = CORT (30 mg kg^−1^), respectively. GA + E1 and GA + E2 are the pretreatments with GA followed by CORT.

**Table 4:** Effect of CORT and GA on IL-4 expression in the rat liver tissue.

Parameter	C	GA	E1	E2	E1 + GA	E1 + GA
Intensity of IL-4 antibody	-	+	++	+++	+	+

**Table 5:** Effect of CORT and GA on the architectural parameters of rat liver.

Liver Architectural Parameters	C	GA	E1	E2	GA + E1	GA + E2
CNH	-	-	++	+++	+	++
CCv	-	-	++	+++	-	+
DH	-	-	++	+++	+	+
DHt	-	-	++	++	+	+
H	-	-	+	++	-	-
ND	-	-	++	+++	+	+
VD	-	-	++	+++	+	+

## Data Availability

The original contributions presented in this study are included in the article/Supplementary Materials. Further inquiries can be directed to the corresponding author.

## Supplementary Materials

The following supporting information can be downloaded at: https://www.mdpi.com/article/10.3390/toxics13100897/s1, Figure S1: Effect of CORT and GA treatment on the expression of IL-4 (panel A), procaspase 3 (panel B), and expression in the liver homogenates of rats. The results are expressed as the mean fold change (*n* = 6). The western blot of IL-4 (31 kDa), procapase-3 (14 kDa), and β-actin (42 kDa) has been illustrated in panel lane 1; 1-control lane 2; GA (50 mg kg-1), lane 3-E1 (15 mg kg^−1^), and lane 4-E2 (30 mg kg^−1^) CORT, respectively. lane 5- GA + E1 and lane 6 6- GA + E2 indicate the pretreatment of animals with GA followed by CORT exposure; Figure S2: Docking studies of Keap 1 with GA, binding energy −7.0; reference compound bardoxolone methyl, binding energy −9.8; Interaction of Ikkb kinase with GA, binding energy −4.8; reference compound Auranofin, binding energy −4.6; (e) Interaction of COX-1 kinase with GA, binding energy −6.1, and reference molecule Diclofenac, binding energy −6.8.

## Abbreviations

The following abbreviations are used in this manuscript:

CCORT	Corticosterone
GGA	Gallic acid
IIL-4	Interleukin-4
kKeap-1	Kelch-like ECH-associated protein 1
IIKKβ	IκB kinase β
CCOX-1	Cyclooxygenase-1
GGCs	Glucocorticoids
