# Supplementary material for: Protective Role of Gallic Acid Against Corticosterone-Induced Hepatic Toxicity: Modulation of Oxidative Stress and Inflammatory Pathways in Wistar Rats
Source: Toxics. 2025 Oct 20;13(10):897. doi: 10.3390/toxics13100897 (PMC12567957; doi:10.3390/toxics13100897)
Supplement: Supplementary file 1 [file toxics-13-00897-s001.zip › toxics-3880814-supplementary.pdf]

Supplementary Materials

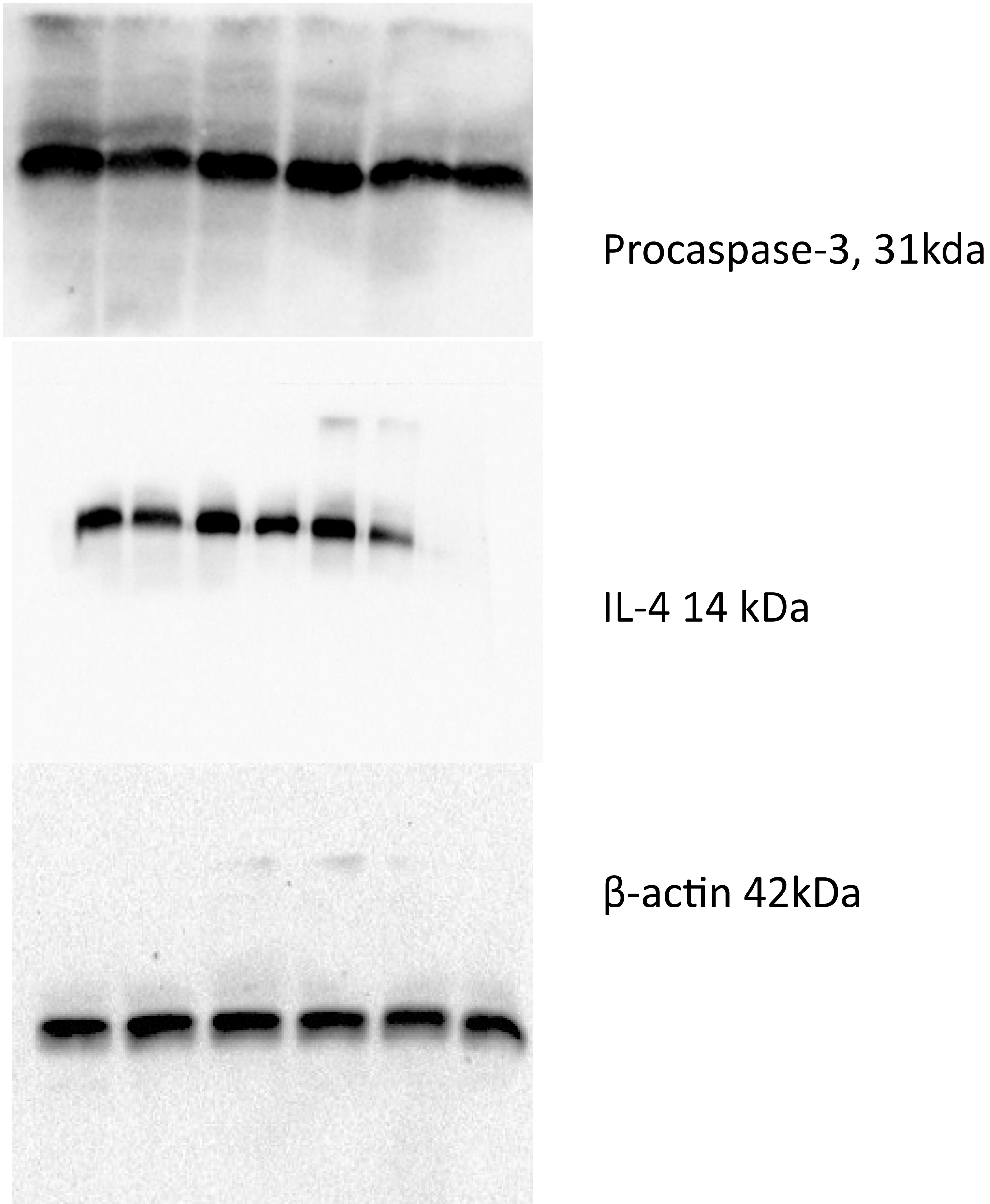

Figure S1 Effect of CORT and GA treatment on the expression of IL-4 (panel A), procaspase 3 (panel B), and expression in the liver homogenates of rats. The results are expressed as the mean fold change (n = 6). The western blot of IL-4 (31kDa), procapase-3(14kDa), and β-actin (42kDa) has been illustrated in panel lane 1; 1-control lane 2 ;GA (50 mgkg^{-1}), lane 3-E1 (15 mg kg^{-1}), and lane 4-E2 (30 mg kg^{-1}) CORT, respectively. lane 5- GA+E1 and lane 6 6- GA+E2 indicate the pretreatment of animals with GA followed by CORT exposure.

Figure S2. Docking studies of Keap 1 with GA, binding energy -7.0; reference compound bardoxolone methyl, binding energy -9.8; Interaction of Ikkb kinase with GA, binding energy

-4.8; reference compound Auranofin, binding energy -4.6; (e) Interaction of COX-1 kinase with GA, binding energy -6.1, and reference molecule Diclofenac, binding energy -6.8.

GA with kaep1

mode | affinity | dist from best mode
| (kcal/mol) | rmsd l.b.| rmsd u.b.
1 3ZGD -7.0 0.000 0.000
spacing 0.375
npts x= 40 y= 40 z= 40
center x = -3.239,y = -9.611 z= 38.493

bardoxolone methyl Kaep1

mode | affinity | dist from best mode
| (kcal/mol) | rmsd l.b.| rmsd u.b.
1 3ZGD -9.8 0.000 0.000
spacing 0.375
npts x= 40 y= 40 z= 40
center x =-3.239 y=-9.611 z=38.493

COX1- gallic acid

mode | affinity | dist from best mode
| (kcal/mol) | rmsd l.b.| rmsd u.b.
1 6Y3C -6.1 0.000 0.000
spacing 0.375
npts x= 40 y= 40 z= 40
center x=-36.654 y= -51.733 z=2.080

COX1- diclofenac

mode | affinity | dist from best mode
| (kcal/mol) | rmsd l.b.| rmsd u.b.
-----+------------+----------+----------
1 6Y3C -6.8 0.000 0.000
spacing 0.375
npts x=40 y= 40 z= 40
center x=-36.654 y= -51.733 z= 2.080

IKK gallic

mode | affinity | dist from best mode
| (kcal/mol) | rmsd l.b.| rmsd u.b.
-----+------------+----------+----------
1 4KIK -4.8 0.000 0.000
spacing 0.375
npts x= 40 = 40 z= 40
center x= 25.359 y= -2.538 z - 85.635

IKK - Auranofin

mode | affinity | dist from best mode
| (kcal/mol) | rmsd l.b.| rmsd u.b.
-----+------------+----------+----------
1 4KIK -4.6 0.000 0.000
spacing 0.375
npts x= 40 y= 40 z= 40
center x= 25.359 y= -2.538 z= - 85.635
